# Changes in temperature, rainfall and snow cover during the reproductive season of Arctic-breeding birds

**DOI:** 10.1007/s00484-026-03348-5

**Published:** 2026-10-16

**Authors:** Nomikos Skyllas, Nicoleta Tsakali, Hans Linssen, Thomas K. Lameris, Bart A. Nolet, Richard Bintanja

**Affiliations:** 1 Energy and Sustainability Research Institute Groningen (ESRIG), University of Groningen, Groningen, the Netherlands; 2 Royal Netherlands Meteorological Institute (KNMI), De Bilt, Utrecht, the Netherlands; 3 Department of Theoretical and Computational Ecology, Institute for Biodiversity and Ecosystem Dynamics, University of Amsterdam, Amsterdam, The Netherlands; 4 Department of Animal Ecology, Netherlands Institute of Ecology (NIOO-KNAW), Wageningen, The Netherlands; 5 Groningen Institute for Evolutionary Life Sciences (GELIFES), University of Groningen, Groningen, the Netherlands

**Keywords:** Arctic tundra, Snowmelt, Phenology, Productive period, Climate projections, CMIP6, Extreme events, Migratory birds, EC-Earth

## Abstract

Arctic warming is rapidly altering hydroclimatic conditions, yet its implications for the timing and reliability of terrestrial breeding environments remain poorly quantified. This study assesses how changes in temperature, snow dynamics, and rainfall extremes affect the snow-free reproductive window across Arctic tundra systems. We combined ERA5 reanalysis (1940–2024), daily output from ten CMIP6 Earth System Models, and long-term equilibrium simulations (400 years) from EC-Earth3 under present-day and doubled CO₂ conditions. Analyses focused on lowland tundra regions and quantified changes in snow cover, timing and duration of the productive period, and the frequency and magnitude of extreme rainfall events. Results show substantial Arctic warming under SSP2-4.5 (approximately 4.9 °C by 2081–2100 relative to 1995–2014), reductions in snowfall, and declining snow cover across the Arctic. Separately, the idealized EC-Earth equilibrium experiment, representing approximately 6 °C of global-mean warming relative to pre-industrial conditions in the 2×CO₂ state, shows how reproductive-season hydroclimate responds under strong sustained warming. Between the 1×CO₂ and 2×CO₂ equilibrium states, the snow-free period lengthens substantially due to earlier snowmelt (–28 days) and delayed snow onset (+36 days), while rainfall extremes intensify sharply. Extremely wet days become up to sixteen times more frequent, and rainfall events with a 100-year return period in the 1×CO₂ climate recur every 2–9 years in the 2×CO₂ climate, with particularly large relative changes in historically dry regions. These results show that a longer snow-free season under strong warming can coincide with substantially greater exposure to rainfall extremes, highlighting the importance of hydroclimatic extremes when assessing ecological responses to Arctic climate change.

**Supplementary Information:**

The online version contains supplementary material available at https://doi.org/10.1007/s00484-026-03348-5.

## Introduction

Arctic migratory birds breed in one of the most challenging environments on Earth. Their entire reproductive cycle depends on a short summer window, the productive period (PP), between spring snowmelt and the return of snow cover (Meltofte et al. 2007). Within weeks, birds must arrive, lay eggs, rear their chicks, and prepare for migration. Any temporal variation in this window, whether from delayed snowmelt, early snow return, or extreme weather, can jeopardize reproductive success if not offset by the bird’s timing (Skinner et al. 1998; Schekkerman et al. 2003). As a result, many Arctic migrants exhibit some degree of flexibility in their migration and breeding phenology, adjusting in response to climatic conditions encountered throughout spring and summer (e.g. Liebezeit et al. 2014; Boom et al. 2023b; Lameris et al. 2018, 2025; Sauve et al. 2019; Linssen et al. 2025).

Climate change is posing challenges to Arctic-breeding birds, by destabilizing the timing of the PP, although current changes such as an earlier snowmelt may in fact be beneficial for these birds (Weiser et al. 2018). The Arctic is warming at least two to three times faster than the global average, with recent assessments indicating rates nearly four times higher since 1979 (Rantanen et al. 2022), though the precise ratio depends on the time period, seasonal window, and geographic definition of the Arctic region analyzed (Serreze and Barry 2011; Gutiérrez et al. 2021). Moreover, the annual mean precipitation is projected to increase by between 17% and 49% by the end of the 21st century, depending on the emissions scenario (Gutiérrez et al. 2021). As long as birds are flexible enough to advance spring migration in synchrony with an earlier start of the PP (but see Linssen et al. 2025; Skyllas et al. 2026), an earlier PP is likely to positively affect reproductive success of migratory birds as earlier onsets of spring are generally associated with higher breeding output, at least in Arctic-nesting geese (Nolet et al. 2020). However, an increase in rain can have strong negative impacts especially for offspring depending on arthropod prey, as rain penetrates downy feathers and can reduce arthropod activity (Oortwijn 2025). The predicted climatic shifts are not uniform: maritime regions are trending toward rain-dominated winters, while continental interiors remain colder but highly variable. For birds adapted to specific local climates, such spatial and seasonal contrasts can be decisive.

Many climate studies, however, do not focus on the variables or spatiotemporal scales most relevant to Arctic–breeding birds. The Arctic is often analysed as a single unit, sometimes encompassing both land and ocean, or summarized in annual means (Sledd and L’Ecuyer 2021; Bintanja and Andry 2017; Screen and Deser 2019). Such approaches overlook the sharp regional and seasonal contrasts that shape ecological opportunities for the organisms that inhabit the Arctic, such as the differences between wet maritime and dry continental regions, or the transitional autumn months when snow cover retreats or returns. Moreover, most ecological studies focus on changes in mean conditions (Bozinovic et al. 2011; Vázquez et al. 2017), whereas weather extremes can also be important. Extreme climatic events, such as unusually persistent snow in spring or strong winds associated with heavy rain in summer, have been reported to trigger complete reproductive failures in Arctic bird species (Schmidt et al. 2019; Yannic et al. 2014). Yet despite their disproportionate impact, extremes and variability remain understudied in Arctic ecology.

Here, we analyse past and future projected climate conditions during the reproductive season of Arctic migratory birds, including the occurrence of precipitation extremes. We focus on terrestrial birds such as waterfowl and shorebirds inhabiting lowland tundra ecosystems, where season length is primarily governed by snow cover. This is in contrast to seabirds, which breed on cliffs and experience a different set of climatic constraints and are therefore outside the scope of this study. Our goal is to provide climate metrics directly relevant to ecology, such as snowmelt dates and extreme rainfall within the PP, and to assess how spatial and seasonal variability may shape future reproductive success of Arctic-breeding birds. We examine four key weather variables: surface temperature, rainfall, snowfall, and snow cover. We investigate how the PP and the occurrence of weather extremes inside that window may change by generating long, steady-state climate simulations with fixed CO₂ forcings (1× and 2× today’s level of 420 ppm). These experiments provide a clean comparison between present-day and a strongly perturbed climate by allowing us to sample numerous extreme events under stable conditions.

To generate our simulations, we combine available long-term reanalysis products from the European Centre for Medium-Range Weather Forecasts (ECMWF) ERA5, an ensemble of Coupled Model Intercomparison Project 6 (CMIP6; Eyring et al. 2016) Earth System Models (ESM), and long equilibrium experiments with the EC-Earth3 ESM (Döscher et al. 2022) to capture both historical trends and future projections. This approach allows us to evaluate and compare changes across the whole Arctic (60–90°N) as well as across ecologically significant subregions, and the different months of the year. In addition to transient scenarios that follow socio-economic pathways, the equilibrium experiments (1× and 2×CO₂) provide stable climates for assessing variability and extremes with high statistical confidence. Finally, we examine how these climatic shifts affect the biologically critical PP, with particular attention to the frequency, timing, and intensity of extreme rainfall events.

Together, these datasets provide complementary perspectives on Arctic climate change. ERA5 describes the recent climate experienced by Arctic-breeding birds and serves as a reference for evaluating the CMIP6 ESMs. The CMIP6 ensemble is used to assess transient changes in temperature, rainfall, snowfall and snow cover over the twenty-first century. The EC-Earth equilibrium simulations address a different question: their long, stable climate states allow us to quantify how the timing of the productive period and the statistical characteristics of rare and extreme rainfall events respond under strong sustained warming. (see "EC-Earth3 equilibrium experiments" section for how this experiment relates to the SSP2-4.5 projections). Together, these approaches allow us to assess both projected changes in Arctic breeding conditions over the coming decades and the response of reproductive-season hydroclimate to strong sustained warming.

## Methods

In the following sections, we describe the three climate datasets used in the study and how they were combined to analyse recent conditions, future projections, and equilibrium responses. We then outline the steps used to derive seasonal and regional climate indicators, including PP timing and extreme events.

### ERA5 reanalysis data

**Fig. 1:**
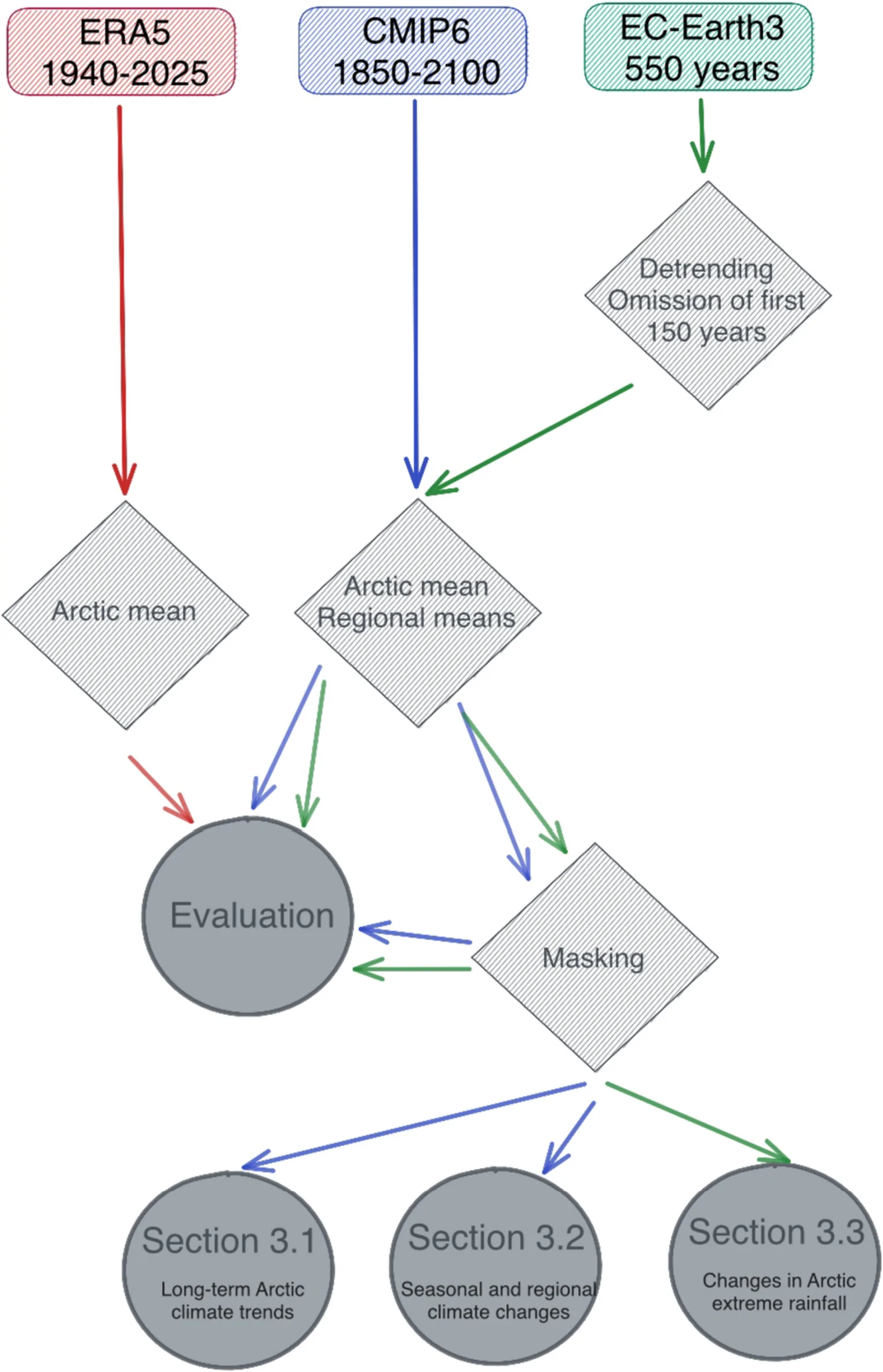
Data sources, processing steps, and analysis workflow. Three datasets were used: ERA5 reanalysis (1940–2024, red), CMIP6 simulations (1850–2100, blue), and EC-Earth3 long steady-state runs (550 years, green). EC-Earth3 data were detrended and the first 150 years omitted before use. For CMIP6 and EC-Earth3, Arctic and regional means were calculated; ERA5 was used for Arctic means only. Masking refers to the sequential application of three spatial masks to retain relevant Arctic land pixels (see "Spatial masks and regional subdivision" section)

**Fig. 2:**
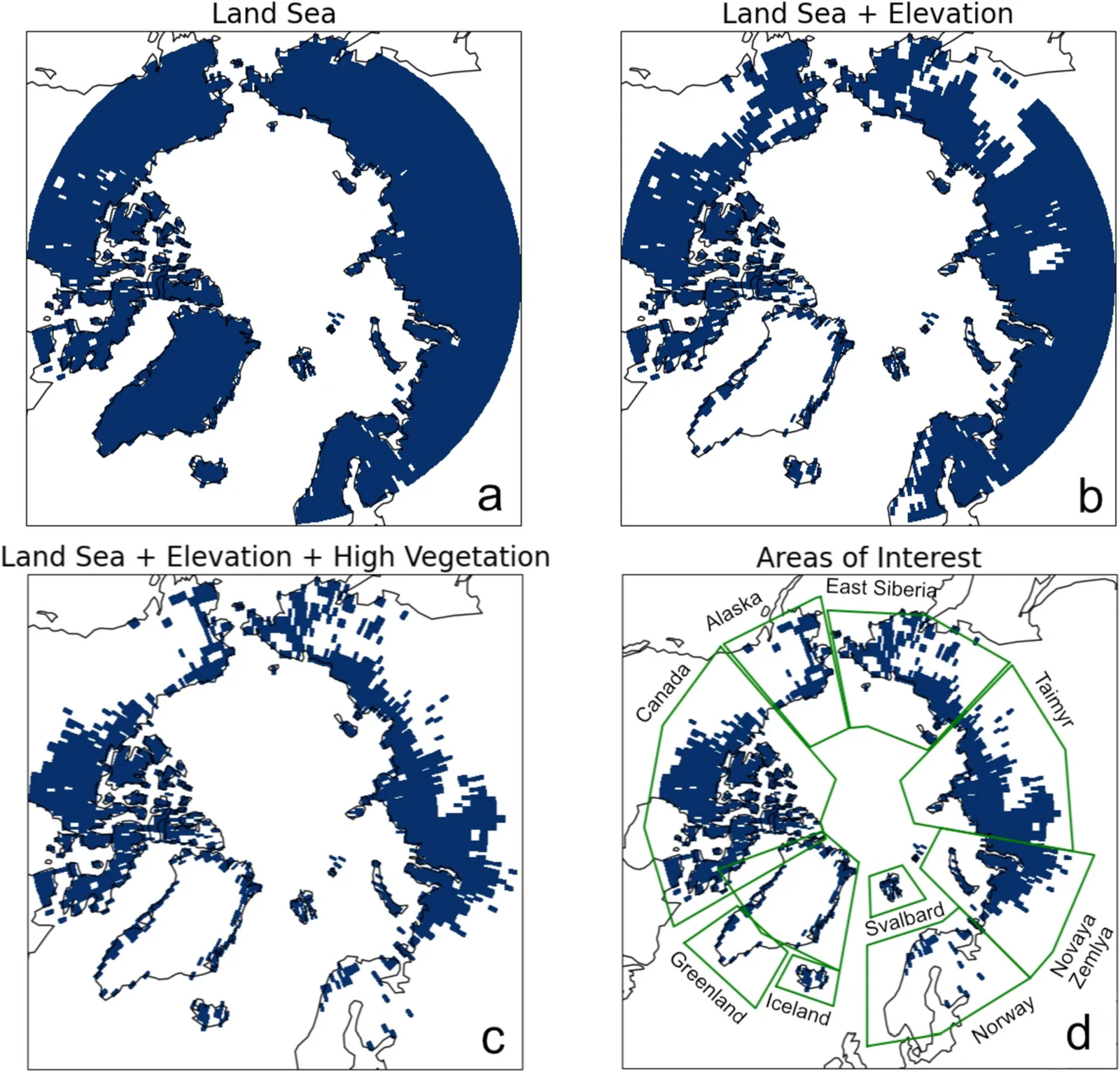
Spatial masks used in the analysis. Sequential application of three spatial masks to retain relevant Arctic land pixels: (**a**) land–sea mask, (**b**) land–sea plus elevation mask (excluding areas > 600 m), and (**c**) land–sea plus elevation plus high-vegetation mask (retaining tundra and other low-vegetation areas). Panel (**d**) depicts the areas selected and used in this study; region names are shown within each area. Greenland is represented by two polygons to capture the relevant Greenland domain while excluding Iceland. The Fennoscandian area is referred to as “Norway” throughout the study because the majority of retained pixels within this area are located in Norway

To characterise recent Arctic climate and provide a baseline for model evaluation (Fig. 1), we used surface air temperature (2 m), total precipitation, and snowfall from the ECMWF Reanalysis v5 (ERA5; Hersbach et al. 2020). Data were downloaded at ~ 25 km spatial resolution (0.25° x 0.25°) and monthly temporal resolution for the period 1940–2024, averaged annually, and regridded to a 1° × 1° grid. The Arctic was defined as the region between 60–90°N (Fig. 2). Because snow cover is not available in the standard ERA5 atmospheric product, we utilized ERA5-Land, a re-run of the land component of the ERA5 reanalysis. ERA5-Land provides a higher spatial resolution (~ 10 km) and is specifically optimized for land-surface variables, ensuring a more accurate representation of terrestrial conditions in complex Arctic topography (Muñoz-Sabater et al. 2021). We obtained snow cover fraction at ~ 10 km resolution for the 1950–2024 period and processed it to match the climate model output for direct comparison.

ERA5 is widely regarded as one of the most reliable reanalysis products for Arctic climate studies and provides the most comprehensive observationally constrained reference currently available for evaluating large-scale hydroclimatic patterns (Cast et al. 2026). We acknowledge that uncertainties are greater before the satellite era (pre-1979) because of sparser observational coverage, but ERA5 remains the most suitable dataset for validating Earth system model behaviour across the pan-Arctic (Gutiérrez et al. 2021; Cast et al. 2026).

### CMIP6 Earth system model simulations

To assess future Arctic climate change (Fig. 1), we analysed daily output from ten CMIP6 Earth System Models (ESMs). We selected three Shared Socioeconomic Pathways (SSPs; O’Neill et al. 2016) to span a broad range of future forcing trajectories: SSP1-2.6 (low), SSP2-4.5 (intermediate), and SSP5-8.5 (high). These scenarios correspond to approximate radiative forcings of 2.6, 4.5, and 8.5 W m⁻² by 2100. SSP5-8.5 represents a very high-emissions pathway and is better interpreted as a high-end rather than a central twenty-first-century scenario (Riahi et al. 2017; Burgess et al. 2023). It is included here only to illustrate the high end of the scenario range and is not used as the basis for our main conclusions, which focus on SSP2-4.5. Detailed regional results across scenarios, including SSP5-8.5, are presented in Supplementary Figure S2. Each SSP simulation was merged with its corresponding historical run (1850–2014), yielding continuous daily output from 1850 to 2100. One ensemble member per model was used. All data were regridded to a common 1° × 1° grid and aggregated to annual resolution for model evaluation.

**Fig. 3:**
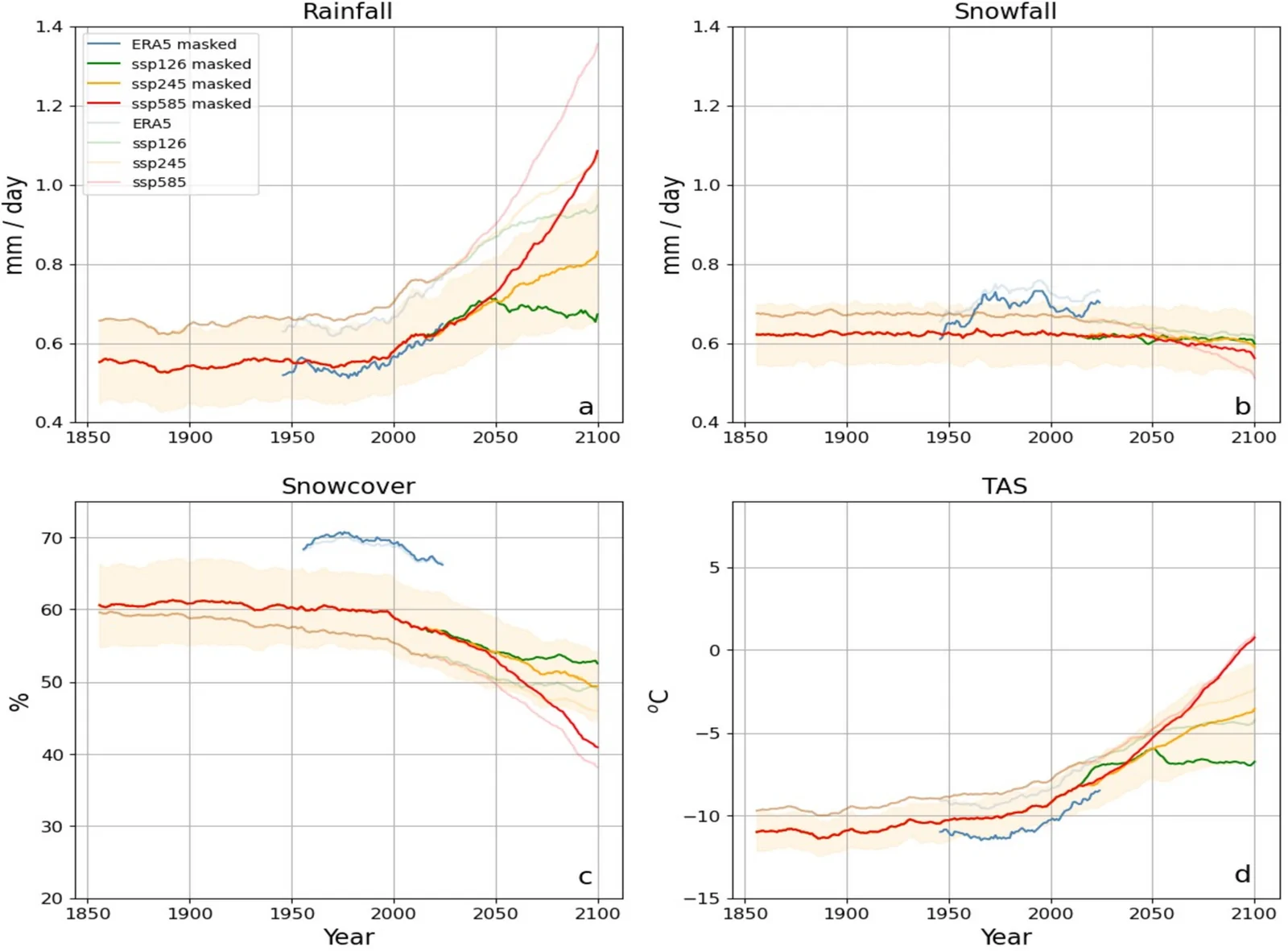
Long-term Arctic mean climate trends (1850–2100) from CMIP6 models (mean over 10 models) and ERA5/ERA5-Land reanalysis. Panels show annual mean (**a**) rainfall (mm water equivalent per year), (**b**) snowfall (mm water equivalent per year), (**c**) snow cover fraction (%), and (**d**) near-surface air temperature (°C). Values represent Arctic-wide averages with a 7-year rolling mean applied to improve visual clarity. Thick lines indicate masked means (means of the area shown in Fig. 2c), while faint lines show unmasked Arctic-wide averages. Reanalysis data are taken from ERA5 for rainfall, snowfall, and air temperature (1940–2024) and from ERA5-Land for snow cover (1950–2024). CMIP6 simulations are shown for SSP1-2.6 (green), SSP2-4.5 (orange), and SSP5-8.5 (red). ERA5 and ERA5-Land are generally consistent with CMIP6 historical simulations up to the early 21st century, supporting the reliability of the model projections. Shading is shown for SSP2-4.5 only and represents the inter-model standard deviation of the annual means across the ten CMIP6 models

Model performance was evaluated by comparing Arctic mean values from the CMIP6 ensemble (ensemble mean across models) against ERA5 over 1940–2024 for temperature, rainfall, and snowfall, and over 1950–2024 for snow cover. Quantitative evaluation (RMSE) was based on the tundra-masked data used throughout the analysis. In addition, comparisons with unmasked Arctic means were examined visually (Fig. 3) to ensure that masking did not introduce systematic biases. The ensemble reproduced the observed Arctic means reasonably well, with RMSE values of 0.98 °C for temperature, 0.02 mm/day for rainfall, 0.09 mm/day for snowfall, and 11% for snow cover.

The ten CMIP6 models were selected based on the availability of all required daily variables (surface air temperature, total precipitation, snowfall and snow cover), which are necessary for deriving productive period timing and extreme-event metrics. The models included were CanESM5, CMCC-CM2-SR5, CMCC-ESM2, INM-CM4-8, INM-CM5-0, IPSL-CM6A-LR, MIROC6, MPI-ESM1-2-HR, MPI-ESM1-2-LR and IITM-ESM. This subset spans a broad range of equilibrium climate sensitivities, including both relatively high-sensitivity (e.g. CanESM5 and IPSL-CM6A-LR) and relatively low-sensitivity (e.g. INM-CM4-8 and INM-CM5-0) models (Sherwood et al. 2020; Hausfather et al. 2022). Models were selected based on data availability rather than climate sensitivity.

Breakpoints in the long-term Arctic climate time series were estimated using piecewise (segmented) linear regression with the piecewise-regression Python package (Pilgrim 2021). The analysis was applied to the Arctic-mean annual time series shown in Fig. 3. Breakpoints represent the estimated years at which the slope of the fitted linear trend changes, indicating a change in the rate of the long-term climate trend.

### EC-Earth3 equilibrium experiments

To quantify climate variability and robustly estimate rare extreme events (Fig. 1), we analysed long equilibrium simulations from the EC-Earth3-CC model configuration. Two fixed-CO₂ experiments were conducted: one at present-day CO₂ levels (1×CO₂, 420 ppm) and one at doubled CO₂ (2×CO₂, 840 ppm). Each experiment was integrated for 550 years under constant forcing (Supplementary Figure S1). Initial conditions were generated from a 100-year spin-up initialized from the SSP2-4.5 simulation. The first 150 years of each equilibrium run were discarded as spin-up, leaving 400 years for analysis. All variables were linearly detrended to remove residual model drift. Daily surface temperature, rainfall, snowfall, and snow cover were analysed, with all output interpolated to a 1° × 1° grid to ensure consistency with ERA5 and CMIP6. These equilibrium simulations represent idealized sensitivity experiments rather than transient future projections. Unlike the SSP simulations, this setup does not correspond to a specific emissions scenario or time period and omits time-varying forcings such as evolving aerosol concentrations, which can substantially influence Arctic climate. We use the long, stable climate states to investigate how productive-period timing and the statistical characteristics of rainfall extremes respond to strong sustained warming. The approximately 10 °C difference in Arctic mean temperature between the 1×CO₂ and 2×CO₂ simulations, measured between the two equilibrium states themselves, represents a substantially stronger warming state than expected under SSP2-4.5 (see "Steady-state runs as a tool for extreme event analysis" section for the corresponding comparison against SSP2-4.5 and pre-industrial baselines) and should not be interpreted as a plausible projection for the end of this century. Instead, the 2×CO₂ simulation provides an idealized strongly warmed equilibrium climate in which changes in these processes and their regional responses can be quantified over a 400-year period.

This setup enables direct estimation of extreme-event statistics without assumptions about stationarity, as long simulations provide sufficient sampling of rare events. In contrast, transient SSP simulations describe a continuously evolving climate system that has not fully equilibrated by 2100 and are therefore less suited for robust extreme-value estimation.

Together, the equilibrium experiments and transient CMIP6 simulations provide complementary.

perspectives: CMIP6 describes the pathway and timing of change, while the equilibrium simulations characterise the magnitude, variability, and extremes associated with a strongly warmed Arctic climate.

### Spatial masks and regional subdivision

Because the study focuses on migratory birds breeding in lowland tundra, all analyses were restricted to this habitat. Without masking, mixed land-cover types (e.g. ocean, mountains, forests) would bias estimates of snowmelt timing, precipitation, and extreme events.

Spatial masks were constructed from ERA5-Land fields and applied consistently to ERA5, CMIP6, and EC-Earth after regridding. ERA5-Land provided land–sea fraction, elevation (from geopotential height), and high-vegetation fraction. Source fields were interpolated to a 1° × 1° grid and cropped to the Arctic domain (60–90°N).

1. Land–sea mask: grid cells with > 50% land fraction were retained. This avoids including mixed coastal/ocean-dominated pixels, which would otherwise strongly influence precipitation and snow cover at this resolution.
2. Elevation mask: Cells below 600 m were retained to capture lowland tundra. Most tundra-breeding shorebirds nest at low elevations, with Johnson et al. 2007 using 350 m as the upper limit for shorebird nesting, whereas for instance, white geese (genus Chen) generally occur below 200 m during the reproductive season (Hughes et al. 1994; Slattery and Alisauskas 2007). A strict 350 m cutoff would be too restrictive at the coarse 1° grid scale (~ 100 km), where many grid cells with mean elevations between 350 and 600 m might still be dominated by low-relief tundra and therefore function ecologically like lowland breeding habitat. The 600 m threshold therefore provides a practical upper limit that includes all low-elevation breeding environments while excluding high-elevation terrain with markedly different snow and precipitation regimes.
3. Vegetation mask: Cells with > 20% high-vegetation cover were excluded. This removes boreal forest and tall shrub zones, which have very different snow and moisture regimes. A 20% threshold retains open tundra including shrub–tundra mosaics, while preventing forest-dominated pixels from influencing climate averages. Lower values (e.g., 10%) would incorrectly exclude many tundra pixels; higher values (e.g., 30%) would allow forested areas to remain.

The combined mask was applied to all daily variables, ensuring that climate metrics reflect conditions relevant to tundra breeding habitats (Fig. 2).

For spatial analyses, the masked Arctic domain was subdivided into nine ecologically relevant regions: Norway, Svalbard, Novaya Zemlya, Taimyr, East Siberia, Alaska, Canada, Greenland, and Iceland (Supplementary Table S1; Fig. 2d). These regions represent major Arctic breeding areas and capture key longitudinal and climatic contrasts. Area-weighted regional means were computed for all datasets and scenarios.

### Seasonal timing and extreme event analysis

#### Productive period timing

We defined the Arctic PP as the annual window between spring snowmelt and autumn snow return, based on daily snow cover fraction. Melt onset was identified as the first day when regional mean snow cover (smoothed by applying a seven-day rolling window) dropped below 50%, and snow return as the first day when snow cover exceeded 30% again. The 50% snow cover is often used to mark the onset of the productive period, as with acceleration of snowmelt the vegetation starts to grow and primary consumers start to become active, providing nest sites and food resources for migratory birds (e.g. Lameris et al. 2018; Oortwijn et al. 2025). Less research has been done into the end of the productive period but the few studies that have looked into this find that departure is initiated when temperatures start to drop below 0 °C (Xu and Si 2019; Solovyeva et al. 2026). A threshold of 50% snow cover would thus overestimate the length of the PP. Setting the threshold too low (e.g. 10% snow cover) may not always work, as snow cover may not drop down to 0% during summer, so we chose 30% that will likely correspond to the first snow fall event. PP length was calculated as the number of days between these two thresholds. All metrics were computed both for the whole Arctic (spatial mean) and for the different Arctic subregions. Interannual variability in PP timing and duration was quantified as the standard deviation across years.

#### Frequency, timing and magnitude of rainfall extremes

Extreme events were defined based on present-day climate conditions. Using the 400-year 1×CO₂ equilibrium simulation, we calculated the 99th percentile of daily rainfall for each month and region, representing the most intense 1% of events under current climate. We then applied these same thresholds to the 2×CO₂ simulation to assess how often present-day extreme events occur in a warmer climate. This allows a direct comparison between present and future conditions, without redefining extremes under changing climate states.

Extreme-event metrics include frequency, intensity, and timing (day-of-year distribution). Return periods were estimated empirically from annual maximum daily rainfall by ranking values across the 400-year simulations. This approach avoids parametric assumptions and allows direct comparison of extreme-event magnitudes between 1× and 2×CO₂ climates. Throughout, “extreme events” refer to extreme daily events.

### Software and data processing

All analyses were performed in Python using xarray (Hoyer and Hamman 2017), numpy (Harris et al. 2020), pandas (McKinney 2010), and piecewise-regression (Pilgrim 2021), alongside Climate Data Operators (CDO; Schulzweida 2021). Processing steps included regridding, spatial masking, temporal aggregation, detrending, percentile and return-period calculations, and regional averaging.

## Results

### Long-term arctic climate trends (1850–2100, CMIP6)

The Arctic climate remained relatively stable until the late 20th and early 21st century (Fig. 3d; surface temperature breakpoint = 2007, rainfall breakpoint = 2020), providing predictable breeding conditions for migratory birds. Since then, rapid shifts in temperature, precipitation, and snow cover have emerged (Fig. 3a–d), coinciding with intensified Arctic amplification (Gutiérrez et al. 2021; Rantanen et al. 2022). Arctic mean rainfall and surface temperature increased sharply in the early 21st century (trends: 10⁻² mm day⁻¹ yr⁻¹ for 2020–2100 and 1.1 × 10⁻¹ °C yr⁻¹ for 2007–2100, respectively), while snowfall remained relatively stable and snow cover declined steadily.

Future projections diverge after ~ 2050. Under SSP1-2.6, Arctic warming levels off, whereas under SSP2-4.5 it continues more gradually than under SSP5-8.5. Under SSP5-8.5, warming accelerates, reaching ~ 8.6 °C Arctic mean warming by 2081–2100 relative to 1995–2014, consistent with CMIP6 assessments (Gutiérrez et al. 2021). Over the same 2081–2100-versus-1995–2014 comparison, Arctic mean warming reaches ~ 4.9 °C under SSP2-4.5 and ~ 2.4 °C under SSP1-2.6.

**Fig. 4:**
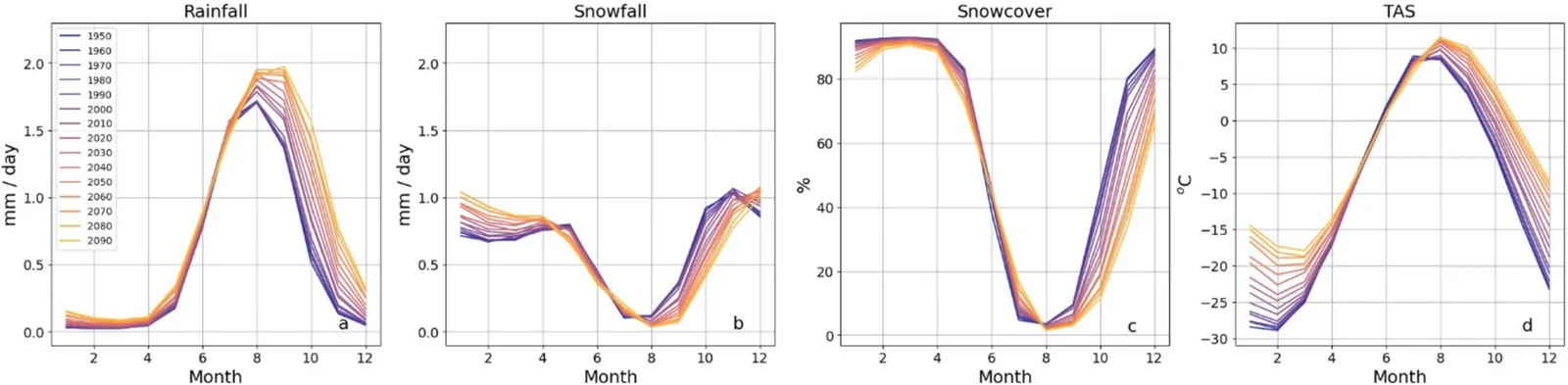
Monthly climatology of the CMIP6 ensemble mean for SSP2-4.5, shown per decade from 1950 to 2090 (line colour from dark blue to yellow). Variables are: (**a**) rainfall (mm/day), (**b**) snowfall (mm/day), (**c**) snow cover fraction (%), and (**d**) 2 m air temperature (°C). Each line represents the multi-model mean monthly values for a given decade, illustrating both long-term trends and shifts in the seasonal cycle

Arctic mean rainfall increases by roughly 33% under SSP2-4.5 (0.6 → 0.8 mm day⁻¹; 2081–2100 mean compared to 1995–2014), while snowfall remains stable or declines slightly, shifting precipitation toward rain-dominated regimes (Fig. 3a, b; Bintanja and Andry 2017). This transition is already emerging in recent observational records; since 1979, annual precipitation has increased, particularly in the Barents Sea sector, accompanied by generally downward trends in snowfall and significant increases in the liquid-to-total precipitation ratio during summer and especially autumn (Cast et al. 2026). By the end of the century, rainfall exceeds snowfall by 33% (SSP2-4.5), indicating a fundamental hydrological transition (Bintanja 2018). Snow cover declines from 58% to 50%, primarily due to reduced autumn and winter snow cover and, to a lesser extent, earlier spring melt (Fig. 4c), shortening the snow season and altering PP timing.

Arctic-wide trends mask strong regional contrasts. In Norway, Iceland, and Svalbard, snowfall decreases throughout the year (0.18–0.43 mm day⁻¹ under SSP2-4.5), driving pronounced reductions in snow cover (11–14%) and a shift towards rain-dominated precipitation (increase of 0.53–0.62 mm day⁻¹, Supplementary Figure S2). In contrast, Canada, East Siberia, and Taimyr retain stable or increasing winter snowfall, yet still experience substantial autumn snow-cover loss as precipitation increasingly falls as rain (autumn rainfall increase of 0.51–0.61 mm day⁻¹). Thus, regional differences extend beyond the magnitude of snow-cover decline: the seasonal timing and the underlying climatic drivers also differ markedly (Supplementary Figs. S2 and S3).

### Seasonal and regional climate changes across the Arctic (CMIP6)

Under SSP2-4.5, Arctic warming is strongest in winter (+ 9.9 °C in 2081–2100 relative to 1995–2014; Fig. 4d), but temperatures remain sufficiently low for precipitation to fall mainly as snow. Total precipitation retains its late-summer maximum, although both the seasonal minimum and maximum shift approximately one month later (Supplementary Fig. S4). Spring and summer warming is more moderate (+ 3.2 °C and + 0.4 °C), with spring precipitation still snow-dominated and limited effects on snow cover (Fig. 4a–c).

In autumn, however, moderate warming (+ 5.9 °C) coincides with a rainfall increase (Fig. 4a; August–December; 0.392 mm/day; same period comparison), delaying snowfall return and shortening the snow season. This shift reflects threshold behaviour: relatively small temperature increases around 0 °C produce abrupt changes in precipitation phase (rain versus snowfall).

Regional patterns reinforce this contrast. Novaya Zemlya, Taimyr, East Siberia, Canada, and Greenland remain cold enough for winter precipitation to remain snow-dominated, partially compensating autumn losses (Cast et al. 2026). In contrast, Svalbard, Norway, and Iceland experience year-round declines in snowfall (Cast et al. 2026), with annual snowfall decreasing by 11–14% (in our projections), driving pronounced winter and spring snow-cover loss. Consequently, the onset of the productive period advances by up to almost two months in these regions (Supplementary Table S2).

### Changes in Arctic extreme rainfall: frequency, timing, and magnitude (EC-Earth steady-state experiments)

Snow defines the start and end of the PP, and hence extreme snowfall plays only a minor role during the PP itself in our 2×CO₂ run (Supplementary Figure S5). Our analysis shows that snowfall extremes occur almost exclusively outside the PP, when birds are absent. By contrast, the absolute occurrence of extreme rainfall events peaks during the PP.

**Fig. 5:**
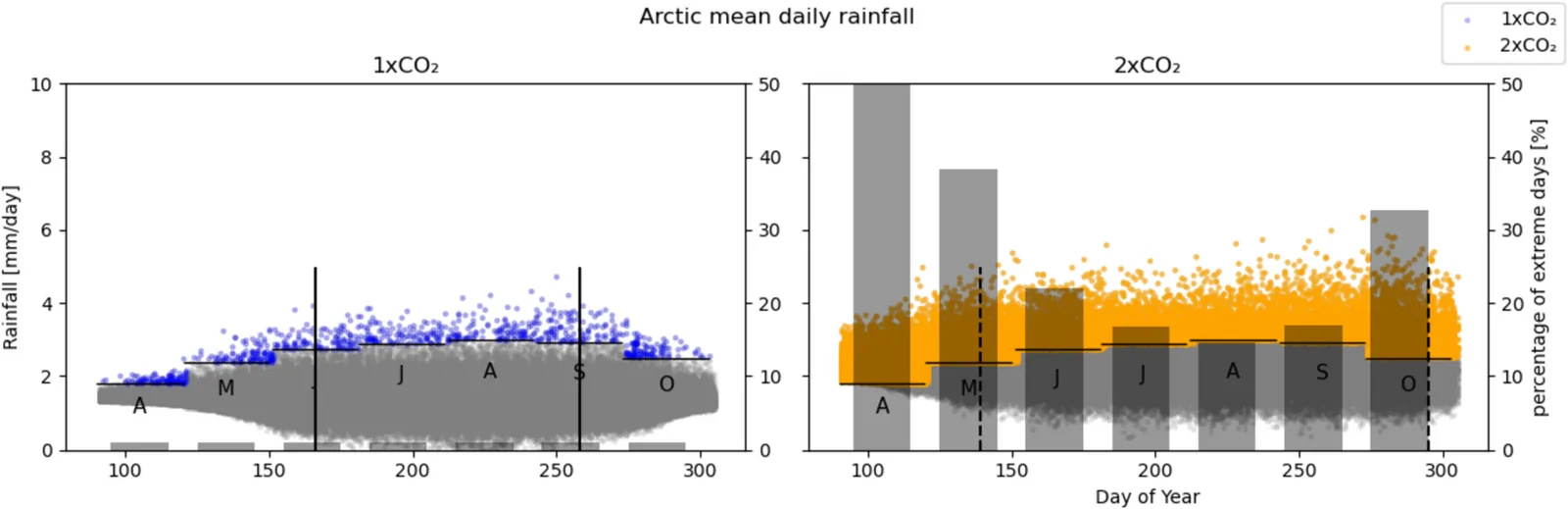
Changes in daily rainfall patterns and extremes in the Arctic under elevated CO₂ levels. Panels show precipitation distributions and extreme rainfall statistics for the Arctic region under present-day (1×CO₂ ≈ 420 ppm) and future (2×CO₂ ≈ 840 ppm) CO₂ levels, based on long-term steady-state simulations. All values are spatially averaged across the Arctic and computed over 400-year time slices per scenario. (**a**) Daily rainfall throughout the year under 1×CO₂, and (**b**) under 2×CO₂. Grey dots show all daily values; blue (**a**) and orange (**b**) dots mark extreme rainfall days, defined as exceeding the monthly 99th percentile from the 1×CO₂ data. Vertical black lines indicate the ecologically relevant productive period (PP), defined by snow cover thresholds: 50% in spring–summer (snowmelt) and 30% in autumn (snow return). Vertical black solid lines mark the PP under 1×CO₂, and vertical black dashed lines mark the PP under 2×CO₂. Overlaid grey bars (right axis) show the proportion of days per month exceeding the 1×CO₂ thresholds (always 1% on the left panel). Horizontal lines represent the monthly 1×CO₂ 99th percentile values. Extremes are defined relative to monthly percentiles to account for seasonal variation. The PP window was determined from modelled snow cover dynamics and applied consistently across both CO₂ scenarios

To investigate how the PP window and (extreme) conditions inside that window may change, we used long, steady-state EC-Earth simulations with fixed CO₂ forcing (1× and 2×). These experiments provide a clean comparison between present-day and a strongly perturbed climate by allowing us to sample numerous extreme events under stable conditions. Our results show that under 2×CO₂ forcing, the PP becomes longer, with snow cover decreasing earlier and returning later. Across the Arctic, snowmelt advances by 28 days (from mean DOY ± standard deviation: 166 ± 4 to 138 ± 3) on average, while snow return is delayed by 36 days (from 259 ± 5 to 296 ± 3, see also vertical lines in Fig. 5), consistent with earlier spring and later autumn temperature threshold crossings (Supplementary Figure S6). While shifts in the timing and duration of the PP are important for migratory birds, the extreme events occurring within this window may be just as important.

It is important to emphasize that the rainfall extremes analyzed here correspond to a climate state that is approximately 7.7 °C warmer than the late-century SSP2-4.5 transient projections discussed in "Long-term Arctic climate trends (1850–2100, CMIP6)" and "Seasonal and regional climate changes across the Arctic (CMIP6)" sections. This figure, together with the related global-mean comparison and the distinct 2×CO₂-versus-1×CO₂ difference reported in "EC-Earth3 equilibrium experiments" section, is discussed in full in "Steady-state runs as a tool for extreme event analysis" section .These results thus illustrate the hydrological limit of a fully equilibrated 2×CO₂ world rather than a prediction for the year 2100.

During the PP1 × (1×CO₂ run, mid-June to mid-September), the frequency of extremely wet days increases ~ 16-fold in the 2×CO₂ experiment compared to the 1× run (Table 1). Outside this window, however, the relative increase compared to present-day conditions is even more dramatic: In October, 32% of all days exceed the 1×CO₂ 99th-percentile threshold (i.e. days that would be classified as “extremely wet” under today’s climate), while in April and May they are even 96% and 38%, respectively. These pre-PP1x months show the most pronounced shifts, reflecting unprecedented extreme rainfall levels and occurrences, driven mainly by higher mean daily precipitation (compared to 1×CO₂, Supplementary Figure S7). Thus, while the PP1x concentrates the largest absolute number of extreme rainfall days, the strongest relative shifts toward unprecedented wet conditions occur outside the PP1x.

Because the Arctic is generally dry compared to most other regions of the world, the threshold for defining an “extremely wet” day is very low. As a result, even modest absolute increases in rainfall translate into large relative changes. This effect is most pronounced outside the PP1x: although rainfall also increases during the PP1x, the much larger ratios in April–May and October indicate that the climate state itself is shifting, with previously relatively dry months experiencing unprecedented extreme rainfall rates. When averaged across study regions (giving each region equal weight), the ratio of extreme wet days during the PP1x remains 3.7 ± 1.2 under 2×CO₂ relative to 1×CO₂ (Table 1).

Regional differences sharpen this contrast. When averaged across the relatively wet regions of Norway, Svalbard, and Iceland (equal weighting of each region), the ratio remains modest (~ 4.5) even when extended to the April-October period, implying that heavier rainfall largely amplifies familiar seasonal patterns. In contrast, the mean across the relatively dry regions of East Siberia, Canada, and Greenland reaches ~ 15.4. Here, modest absolute changes produce disproportionately large relative shifts, creating rainfall regimes with no modern analogue. For migratory Arctic-breeding birds, this means that those species that are accustomed to “wet” regions will encounter stronger rainfall within familiar patterns, whereas birds in historically dry regions will be confronted with a transition toward wetter climates, exposing them to novel and potentially disruptive conditions.

**Table 1:** Increase in the number of extreme rainfall events under doubled CO₂ (2×CO₂) conditions across Arctic regions. Values represent the ratio of days with extreme precipitation (above the monthly 99th percentile of the 1×CO₂ climate) in the 2×CO₂ scenario compared to 1×CO₂. For the PP1x (Productive Period under 1×CO₂ climate) column, extreme days were only counted within each region’s reproductive season, defined by the region-specific snowmelt and snow return dates (days of year when snow cover drops below 50% and rises above 30%, respectively). The 7-month column includes all region-specific extreme events from April to October. High ratios indicate strong intensification of extreme rainfall. More detailed information is presented in Supplementary Figure S8

Region	2x/1x Ratio during PP_1x_	2x/1x Ratio during Apr-Oct
Arctic	15.9	33.7
Norway	3.0	4.1
Svalbard	3.2	7.0
Novaya Zemlya	3.1	5.9
Taimyr	4.3	11.1
East Siberia	4.0	9.6
Alaska	3.1	4.2
Canada	4.7	19.7
Greenland	5.9	17.1
Iceland	2.1	2.5

**Fig. 6:**
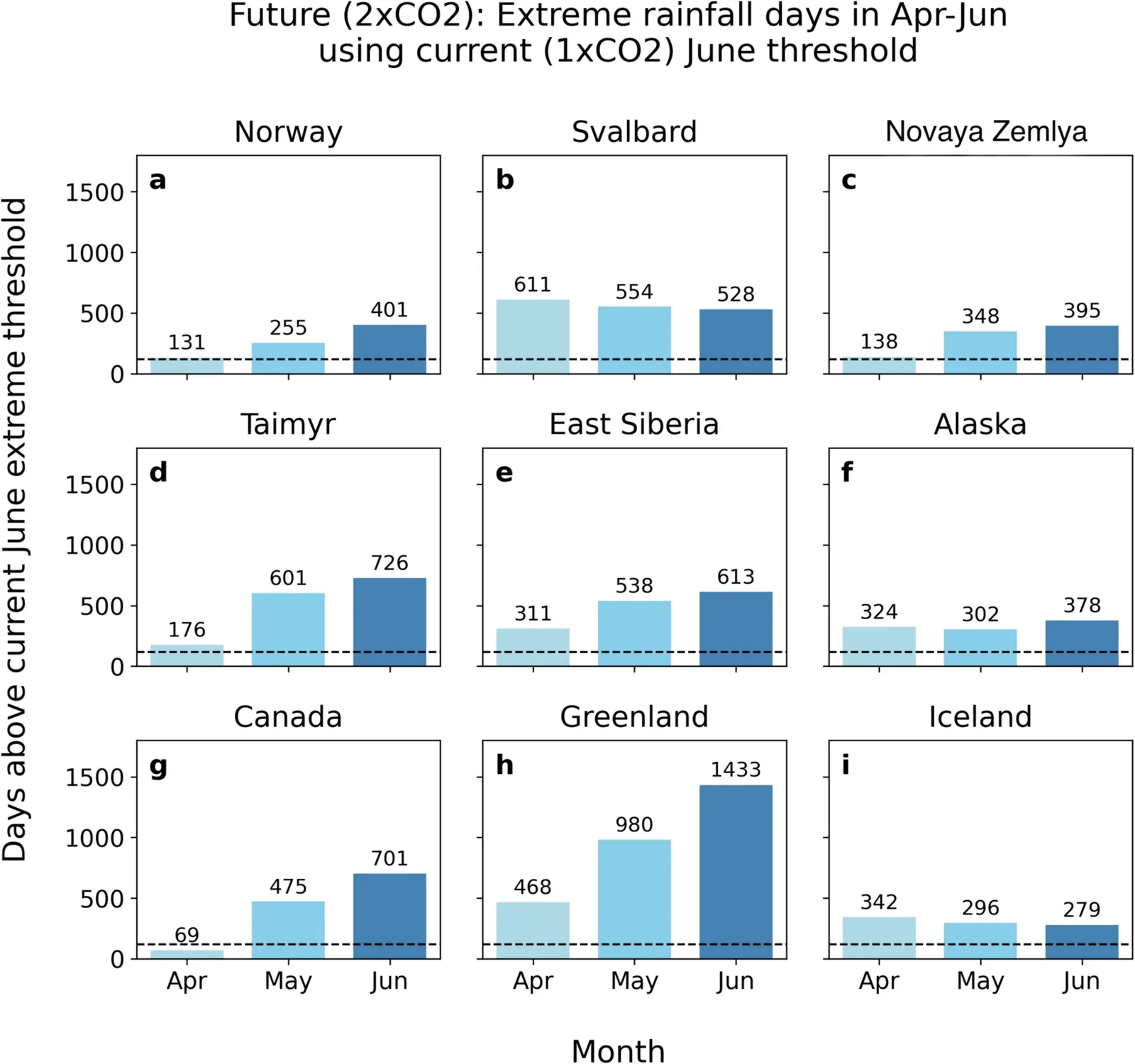
Number of days exceeding the present-day (1×CO₂) June extreme rainfall threshold under future 2×CO₂ conditions, shown for April–June across nine Arctic regions: a) Norway, b) Svalbard, c) Novaya Zemlya, d) Taimyr, e) East Siberia, f) Alaska, g) Canada, h) Greenland and i) Iceland. Bars indicate monthly counts over the 400-year simulations. The dashed line indicates the 1×CO₂ reference frequency (120 days), which is identical across regions because thresholds were defined as the regional June 99th percentile over the 400-year simulation. This illustrates how rainfall extremes spread into earlier months under warming, reducing the potential for migratory birds to avoid extreme conditions by arriving earlier in the season. April is shown to illustrate climatic spillover relative to present-day June, not direct bird exposure

Using June as a reference month, as this marks the period when most Arctic-breeding tundra birds are nesting, we find that under doubled CO₂, there are clear regional differences in how present-day June rainfall extremes change and redistribute seasonally (Fig. 6). Regions such as Greenland and Canada, which have relatively low present-day thresholds, show a strong amplification of June extremes, but limited spillover into May. By contrast, regions where thresholds are already high, such as Iceland and Norway, show smaller relative increases in June itself, but a more even redistribution of June-like extremes into May, and to a lesser extent April. While April primarily reflects a shift in the climatic regime rather than direct exposure of breeding birds, it illustrates how present-day June conditions emerge progressively earlier in the calendar year.

Svalbard shows a mixed response, combining a strong increase in June extremes with pronounced seasonal spillover. Together, these patterns indicate that regions differ in whether climate change primarily intensifies extremes during the core breeding period or redistributes them toward earlier parts of the season once snow-free conditions are reached.

**Table 2:** Return levels of rare and extreme daily rainfall under 1×CO₂ (420 ppm) and 2×CO₂ (840 ppm) climates across nine Arctic regions. For each return period (1-year annual maximum, 10-year, and 100-year), columns report the magnitude under 1× conditions, the magnitude under 2× conditions, and the relative change (Δ, %). Rainfall values are given in mm day⁻¹. Return levels were derived directly from 400-year simulations per scenario (no parametric extreme value fitting). The 10-year and 100-year levels correspond to the 40th and 4th highest values, respectively. The final column gives the return period (years) of the present-day (1×) 100-year event under 2× conditions. More detailed information is presented in Supplementary Figure S9

Region	1-yr return event (mm/day)	10-yr return event (mm/day)	100-yr return event (mm/day)	Return period (1x_100 year) in 2x world (years)
Norway	6.2 -> 8.4 (35%)	13.4 -> 17.1 (28%)	16.5 -> 21.7 (32%)	7
Svalbard	4.3 ->9.2 (114%)	15.8 -> 25.5 (61%)	21.3 -> 34.9 (64%)	4
Novaya Zemlya	5.1 -> 6.2 (22%)	10.0 -> 13.2 (32%)	12.3 -> 15.6 (27%)	6
Taimyr	3.5 -> 5.3 (66%)	6.9 -> 9.2 (33%)	9.0 -> 10.8 (20%)	8
East Siberia	3.2 -> 5.1 (59%)	8.8 -> 12.5 (42%)	12.1 -> 17.2 (42%)	9
Alaska	5.4 -> 7.2 (33%)	14.7 -> 19.7 (34%)	18.1 -> 26.3 (45%)	6
Canada	2.6 -> 3.7 (42%)	5.9 -> 8.4 (42%)	7.3 -> 11.2 (53%)	4
Greenland	2.5 -> 5.3 (112%)	6.4 -> 10.4 (62.5%)	8.0 -> 13.4 (67.5%)	2
Iceland	12.9 -> 15.6 (21%)	27.6 -> 35.3 (28%)	33.8 -> 51.5 (52%)	7

Even if birds were to arrive earlier to avoid the higher frequency of extremes, they would still face more intense downpours than in the past, meaning that months with fewer exceedances may still expose them to intense rainfall levels (Table 2). All regions are projected to experience substantial increases in extreme rainfall events, though the magnitude differs: some, such as Iceland and Norway, show relatively moderate growth, while others, including Canada and East Siberia, face much stronger intensification.

Two regions stand out as extreme cases. Greenland shows the most dramatic change, with the 1-year return event (an event expected roughly once per year) increasing by + 112% and reducing the recurrence time of a present-day 100-year event to only two years. Svalbard displays similarly large proportional increases (+ 114% at the 1-year level and + 64% at the 100-year level), but with absolute rainfall amounts nearly double those projected for Greenland. Taken together, these results imply that rainfall events that are currently rare even within a bird’s whole lifetime, may in the future occur multiple times in a single reproductive season (Supplementary Figure S10).

## Discussion

### Steady-state runs as a tool for extreme event analysis

We used long steady-state (equilibrium) simulations, run under fixed CO₂ concentrations (EC-Earth dataset), to analyse how increasing weather extremes may affect the breeding season of Arctic migratory birds. Unlike transient CMIP6 scenarios, which combine long-term trends with evolving variability (Fig. 3), our simulations hold the background climate constant and allow hundreds or even thousands of years of variability to be sampled under both present-day and perturbed conditions. This design enabled us to separate internal variability from long-term change (van der Linden et al. 2017) and to characterize extreme events with greater statistical confidence than transient simulations permit (Rugenstein et al. 2019).

For our purposes, the steady-state framework was crucial for sampling rare events with high statistical confidence. The equilibrium simulations and the transient CMIP6 scenarios describe different climate states, however, and the warming levels involved differ depending on which baseline and which spatial domain, global or Arctic, is used for the comparison.

In global-mean terms, relative to pre-industrial conditions, the 2×CO₂ equilibrium experiment represents approximately 6 °C of warming, compared with approximately 3.5 °C in EC-Earth3-CC under SSP2-4.5 by 2081–2100 — a gap of roughly 2.5 °C at the global scale. Because Arctic warming is amplified relative to the global mean, this same gap is substantially larger when expressed as an Arctic-mean difference: the 2×CO₂ equilibrium climate is approximately 7.7 °C warmer than the late-century SSP2-4.5 climate in the Arctic (Changes in Arctic extreme rainfall: frequency, timing, and magnitude (EC-Earth steady-state experiments)") section. This 7.7 °C figure and the approximately 10 °C difference between the 1×CO₂ and 2×CO₂ equilibrium states reported in   "EC-Earth3 equilibrium experiments" section describe two distinct comparisons — the former against transient late-century SSP2-4.5, the latter between the two equilibrium states themselves — and are not directly comparable without a common reference point; both nonetheless place the 2×CO₂ experiment well outside the range reached under SSP2-4.5 this century.

A climate state with warming of the magnitude represented by the 2×CO₂ experiment is unlikely to be reached during the twenty-first century under current emissions trajectories. SSP5-8.5, which produces similarly high-end warming in CMIP6, represents a very high-emissions pathway requiring sustained fossil-fuel growth and is better interpreted as a high-end rather than a central twenty-first-century scenario (Riahi et al. 2017; Burgess et al. 2023). Intermediate forcing pathways are more consistent with recent emissions trends (Pedersen et al. 2021). This discrepancy reflects both the different forcing levels represented by the two experiments and the distinction between transient and equilibrium climate responses, in which slow feedbacks have had sufficient time to emerge (Sherwood et al. 2020).

In addition, the equilibrium analysis is based on a single climate model (EC-Earth3-CC), which has a relatively high equilibrium climate sensitivity (Hausfather et al. 2022), and equilibrium warming patterns may differ from those in transient scenario simulations because of the pattern effect (Sherwood et al. 2020). Its value lies instead in the long, stable climate states it provides, which allow changes in productive-period timing and the statistical characteristics of rare rainfall events to be quantified with a precision that transient, non-stationary SSP simulations cannot match. The experiment informs how these processes respond under strong sustained warming, but not when, or along which emissions pathway, changes of this magnitude might be reached.

### Spatial and seasonal variability in Arctic futures

Warming patterns across the Arctic are not uniform. Under SSP2-4.5, relative to 1995–2014 (as in "Long-term Arctic climate trends (1850–2100, CMIP6") section, traditionally cold regions with mean annual surface temperatures below 0 °C show the strongest increases (3.7–5.3 °C by the end of the century), whereas Norway and Iceland, which were already above freezing, warm more moderately (~ 3 °C, Supplementary Figure S2d and ag). Yet even these smaller increases are sufficient to shift a substantial fraction of precipitation from snow to rain, leading to sharp snowfall declines and large reductions in snow cover (~ 12%). This illustrates that even modest warming in relatively mild regions can produce disproportionately large changes in the snow regime.

Svalbard represents an important outlier. Situated at the historical edge of sea-ice extent, it combines features of both colder and milder regions from our study. While its mean annual TAS remains below 0°C despite a warming of 6.1°C, it shows a strong year-round shift in precipitation, with autumn snowfall decreasing by roughly 70% and no compensation by increasing winter snowfall. At the same time, rainfall rises across all seasons (0.26–1.10 mm day⁻¹), driving marked snow cover loss throughout the year (-8 to -21%). The case of Svalbard highlights the role of sea ice in Arctic climate: once protected by the seasonal ice edge, it is now transitioning toward a regime more comparable to Norway and Iceland, with rain increasingly replacing snow and snow cover progressively diminishing (Atlantification). The ‘Atlantification’ of the Barents Sea sector is already evidenced by significant upward trends in annual precipitation north of the Svalbard archipelago (Cast et al. 2026). This shift is increasingly driven by winter sea ice loss, which allows warm Atlantic water to trigger convective-type precipitation that is moving into progressively higher latitudes as winter sea ice retreats (Cast et al. 2026). In this sense, Svalbard may offer a glimpse of the future trajectory of traditionally colder Arctic regions. Although snow cover is declining, the overlap of persistent snow with rising rainfall means that rain-on-snow events are expected to become more frequent and severe, creating ice crusts that limit food access for herbivores (Rennert et al. 2009) and decrease the insulating effect of snow for animals wintering under the snow (Kausrud et al. 2008). Such events may have knock-on effects on the whole food web, including breeding birds (Layton-Matthews et al. 2023; Nolet et al. 2013).

Seasonal variations are equally important. Winter exhibits the strongest warming signal across the Arctic, reflecting the retreat of sea ice at its maximum seasonal extent and the full force of Arctic amplification. Despite this, most regions remain cold enough to sustain snowfall, and enhanced atmospheric moisture can even produce increasing winter snowfall (Supplementary Figure S3n, r, z and ad). In areas such as Greenland, this thicker snowpack may delay snow cover loss, thereby sustaining the traditional timing of the PP. Spring and summer warming is more moderate, but in regions without winter snowfall increases (e.g. Svalbard), snow cover reduction accelerates, advancing the PP onset.

Autumn is found to be the most critical season of change. Even relatively modest warming pushes temperatures above freezing, reduces snowfall, increases rainfall, and delays the establishment of snow cover. At the same time, sea ice is at its seasonal minimum, and delayed snowfall coupled with warmer conditions postpones freeze-up, enhancing ocean–atmosphere heat fluxes and further amplifying Arctic warming and wetting. Although migratory birds typically depart during this season, autumn processes set the stage for the subsequent winter and spring. Later snow onset and delayed sea-ice formation can cascade into warmer winters and earlier spring melt, shortening the snow season.

Taken together, these results show that both spring advances and autumn delays shape the timing of the PP, but the balance between them differs regionally. Relatively cold regions are projected to remain snow-dominated in winter, preserving a relatively short but still snow-governed reproductive window. By contrast, regions near the sea-ice edge, such as Svalbard, are shifting toward longer yet wetter seasons, where snow cover is reduced and rainfall plays a growing role. For birds, this implies divergent risks: some populations will continue to face narrow windows constrained by snow, while others will contend with longer seasons increasingly dominated by rain. Ultimately, while some regions currently show winter snowfall increases, this resilience has limits: continued warming is likely to erode it, pushing even the most stable regions toward trajectories resembling Svalbard, with year-round snow loss and rain-dominated precipitation regimes.

### Implications of intensifying extremes for bird reproduction and migration strategies

A longer PP is generally expected to benefit birds by providing more time for reproduction. In waterfowl, earlier and warmer springs can increase the proportion of individuals attempting to breed and improve nesting success (Boom et al. 2023b). Increased rainfall may also have positive effects by enhancing vegetation growth, particularly graminoids, thereby increasing food availability for Arctic-nesting waterfowl (van der Kolk et al. 2016). However, rainfall can also have negative effects on reproduction, especially during early life stages. Young chicks have limited insulation, and prolonged wet conditions can increase brooding time at the expense of foraging, reducing growth rates and survival (Oortwijn 2025). At the nest level, heavy rainfall has been linked to nest failure (Skinner et al. 1998) and chick mortality (Anctil et al. 2014). Moreover, rainfall effects may be indirect and context-dependent: for example, higher summer precipitation in Svalbard barnacle geese was associated with increased fledgling mortality, potentially through increased cloud cover reducing plant productivity (Layton-Matthews et al. 2023). Finally, a later end of the PP may again benefit some waterfowl species, which typically depart the Arctic only as cold conditions set in (Solovyeva et al. 2026) and day length decreases (Boom et al. 2023a), whereas many shorebirds depart breeding grounds shortly after brood-rearing, often as early as mid-July (Duijns et al. 2019).

The EC-Earth equilibrium experiments illustrate strong regional differences in the extent to which earlier breeding could reduce exposure to extreme rainfall during the snow-free period. In the colder continental regions (Novaya Zemlya, Taimyr, East Siberia and Canada), April under the idealised 2×CO₂ climate exhibits frequencies of extreme rainfall events similar to those occurring during June under the 1×CO₂ equilibrium climate (Fig. 6). However, in these same regions the onset of the productive period advances by only 25–37 days (Supplementary Table S2). For example, snowmelt advances from day-of-year (DOY) 166 to DOY 140 in Canada (26 days earlier), and from DOY 172 to DOY 145 in Taimyr (28 days earlier). Within these equilibrium climate states, the productive period therefore begins around May rather than April, suggesting that birds could not fully track the earlier emergence of rainfall extremes because snow-free nesting habitat would generally not yet be available. By May, however, rainfall extremes relative to June under the 1×CO₂ equilibrium climate have already become common (e.g. 475 days in Canada and 601 days in Taimyr over the 400-year simulations; Fig. 6). These experiments therefore suggest that, in continental regions, earlier snowmelt may provide some opportunity for earlier breeding but may not fully offset the earlier seasonal emergence of extreme rainfall.

In contrast, maritime regions such as Svalbard and Iceland show a different response. Here, snowmelt advances by 41–54 days (Supplementary Table S2), yet the earliest snow-free portion of the productive period already experiences frequencies of extreme rainfall higher than those occurring during June under the 1×CO₂ equilibrium climate (Fig. 6). Within these idealised climate states, advancing breeding would therefore be expected to provide only limited reductions in exposure to heavy rainfall because rainfall extremes emerge almost immediately after snow retreat.

Although many Arctic-breeding birds can advance arrival and egg-laying by days or weeks through faster migration (Lameris et al. 2025; Linssen et al. 2025), breeding remains constrained by the availability of snow-free nesting habitat (Liebezeit et al. 2014; Lameris et al. 2018; Boom et al. 2023b). Additional advances beyond snowmelt would require earlier fuelling and departure from non-breeding areas (Linssen et al. 2025), and it remains uncertain whether migratory schedules can fully track the hydroclimatic changes illustrated by these equilibrium experiments (McNamara et al. 2011; Burnside et al. 2021).

The ecological consequences of these changes are likely to depend on baseline conditions, defined here as the present-day rainfall regimes to which each population is adapted. Birds breeding in historically wetter regions (with high extreme rainfall magnitude), such as Norway or Svalbard, already experience frequent rainfall under 1×CO₂ and may tolerate moderate increases under 2×CO₂. In contrast, birds breeding in historically drier regions (with low extreme rainfall magnitude), such as Canada and Greenland, face much larger proportional increases in extreme rainfall frequency and intensity when moving from 1× to 2×CO₂, even though absolute magnitudes remain lower than in wetter regions (Table 2; Supplementary Figure S8). Whether these newly emerging extremes will have comparable ecological impacts remains uncertain: they may overwhelm populations adapted to low-rainfall environments, or alternatively be less damaging than smaller proportional changes in already wet regions. An additional possibility is that shifts in precipitation regimes may modify environmental filtering across regions, potentially allowing species adapted to wetter conditions to occupy areas that were previously too dry. Together, these contrasting responses highlight that both the timing of extreme rainfall within the snow-free period and the ecological context of local adaptation are critical for understanding climate-change impacts on Arctic-breeding birds. 

## Supplementary Information

Below is the link to the electronic supplementary material.

Supplementary Material 1

## Data Availability

The data supporting the results of this study are available via Zenodo at:https://doi.org/10.5281/zenodo.18282287. Raw CMIP6 climate model data used in this study are publicly available through the Earth System Grid Federation (ESGF) portal (https://esgf-node.llnl.gov/). Raw ERA5 reanalysis data are publicly available from the Copernicus Climate Data Store (https://cds.climate.copernicus.eu/datasets). The raw Alternative Earth Variability (ALEVA) data, consisting of 550-year steady-state EC-Earth3 climate model simulations, are not publicly available because they are stored on the ECMWF High Performance Storage System, which is accessible only to ECMWF member states. The processed ALEVA-derived datasets used in this study are available via Zenodo.

## Code Availability

The scripts supporting the results in this paper are available at Zenodo: https://doi.org/10.5281/zenodo.18281938
