# Supplementary material for: Changes in temperature, rainfall and snow cover during the reproductive season of Arctic-breeding birds
Source: Int J Biometeorol. 2026 Oct 16;70(11):280. doi: 10.1007/s00484-026-03348-5 (PMC13675148; doi:10.1007/s00484-026-03348-5)
Supplement: Supplementary file 1 [file 484_2026_3348_MOESM1_ESM.docx]

# Supplementary material for:

# Changes in temperature, rainfall and snow cover during the breeding season of Arctic-breeding birds

##### Supplementary Figure S1: Arctic annual mean near-surface air temperature from two 550-year EC-Earth3 equilibrium simulations with fixed CO₂ forcing: present-day (1×, 420 ppm; blue) and doubled (2×, 840 ppm; orange). Light lines show the raw, unmasked 550-year runs (60–90°N, non-detrended), while dark lines show the masked and linearly detrended 400-year segments used for analysis after discarding the initial 150-year spin-up. All values are annual means, with a 10-year rolling mean applied for visualization clarity only (all calculations are based on unsmoothed annual data). The raw series illustrate the full adjustment and model drift, whereas the masked & detrended series represent the equilibrated climates used in this study: -5.7 °C (raw data: –4.2 °C) under 1×CO₂ and 3.9 °C (raw data: +5.5 °C) under 2×CO₂. These equilibrated states provide the contrasting baselines for our extreme-event analyses, allowing us to assess how a ~10 °C shift in mean Arctic climate alters rainfall extremes, snow cover, and the productive period critical for migratory birds.

##### Supplementary Table S1: Arctic study regions with their bounding coordinates (longitude and latitude ranges).

| Area | Lon min | Lon max | Lat min | Lat max |
| --- | --- | --- | --- | --- |
| Arctic | -180 | 180 | 60 | 90 |
| Norway | 0 | 45 | 57 | 71 |
| Svalbard | 2 | 35 | 75 | 81 |
| Novaya Zemlya | 45 | 57 | 57 | 79 |
| Taimyr | 80 | 136 | 60 | 83 |
| East Siberia | 137 | -169 | 60 | 77 |
| Alaska | -88 | -140 | 58 | 77 |
| Canada | -139 | -60 | 58 | 83 |
| Greenland | -70 | -10 | 67 | 83 |
| Iceland | -28 | -10 | 62 | 67 |

##### Supplementary Figure S**2**: Long-term climate trends (1850–2100) from CMIP6 models for nine Arctic regions. Panels show annual mean a) rainfall (mm water equivalent per year), b) snowfall (mm w.e. per year), c) snow cover fraction (%), and d) near-surface air temperature (°C). Each row corresponds to a different region (Norway, Svalbard, Novaya Zemlya, Taimyr, East Siberia, Alaska, Canada, Greenland, Iceland). Simulations are shown for SSP1-2.6 (green), SSP2-4.5 (orange), and SSP5-8.5 (red). While all regions exhibit warming and snow cover decline, the balance between rainfall and snowfall varies substantially, reflecting strong spatial contrasts.

##### Supplementary Figure S3: Monthly climatology of the CMIP6 ensemble mean for SSP2-4.5, shown per decade from 1950 to 2090 (line colour from dark blue to yellow), for nine Arctic regions. Panels show a) rainfall (mm/day), b) snowfall (mm/day), c) snow cover fraction (%), and d) 2 m air temperature (°C). Each line represents the multi-model mean monthly values for a given decade. Regional patterns highlight long-term warming and a seasonal shift from snow to rain, occurring earlier in maritime regions while persisting longer in continental interiors.

##### Supplementary Figure S**4**: Monthly climatology of Arctic mean total precipitation from the CMIP6 multi-model ensemble under SSP2-4.5, shown for successive decades from 1950 to 2090 (line colours progress from dark blue to yellow). Each line represents the decadal mean monthly precipitation, illustrating long-term changes in precipitation magnitude and shifts in the timing of the seasonal cycle.

##### Supplementary Table S2. Snow-cover-defined productive-period limits under present-day (1xCO2) and doubled-CO2 (2xCO2) climate conditions. Values show the mean day of year (DOY) of snowmelt, defined as the first day when 7-day-smoothed snow cover falls below 50%, and snow return, defined as the first subsequent day when snow cover rises above 30%. Productive-period length is calculated as the interval between snowmelt and snow return. Snowmelt advancement and snow return delay indicate the change in timing under 2xCO2 relative to 1xCO2, with negative onset values indicating earlier snowmelt and positive return values indicating later snow return.

| region | Snowmelt 1x (DOY) | Snow return 1x  (DOY) | Snowmelt 2x  (DOY) | Snow return 2x  (DOY) | Snowmelt advancement  (days) | Snow return delay  (days) |
| --- | --- | --- | --- | --- | --- | --- |
| Norway | 145.31 | 269.36 | 103.02 | 303.75 | -42.29 | 34.39 |
| Svalbard | 173.3 | 225.34 | 118.86 | 294.71 | -54.44 | 69.37 |
| Novaya Zemlya | 160.91 | 275.39 | 123.56 | 303.8 | -37.35 | 28.41 |
| Taimyr | 172.48 | 262.27 | 144.53 | 293.62 | -27.95 | 31.35 |
| East Siberia | 165.54 | 261.18 | 140.95 | 293.17 | -24.59 | 31.99 |
| Alaska | 148.63 | 240.51 | 119.42 | 287.05 | -29.21 | 46.54 |
| Canada | 165.6 | 246.5 | 139.9 | 296.76 | -25.7 | 50.26 |
| Greenland | 183.27 | 219.46 | 151.98 | 279.46 | -31.29 | 60 |
| Iceland | 140.04 | 229.62 | 99.42 | 249.28 | -40.62 | 19.66 |

##### Supplementary Figure S5: Regional daily snowfall distributions and extreme-event statistics under 1×CO₂ (left) and 2×CO₂ (right). Results are shown for nine Arctic regions, each based on 400-year simulations. Grey dots show all daily values; coloured dots mark extreme snowfall days (monthly 99th percentile from the 1× baseline). Grey bars show the proportion of days above threshold. Extreme snowfall events decline under 2×CO₂, nearly disappearing in maritime regions and decreasing substantially elsewhere.

##### Supplementary Figure S6: Arctic mean daily surface temperature under 1×CO₂ (left) and 2×CO₂ (right). Grey dots show all daily values; blue/orange indicate daily means. Vertical black lines mark the PP, with dashed lines showing the alternate scenario for comparison. Shaded grey bands highlight months. 2×CO₂ conditions produce warming across all months, with spring crossing freezing earlier and autumn later, lengthening the snow-free season.

##### Supplementary Figure S7: Arctic mean rainfall (mm/day) for the snow-free season (April–October) under 1×CO₂ (blue) and 2×CO₂ (orange) steady-state simulations. Shaded areas show the interannual standard deviation. Vertical black lines mark the start and end of the productive period (PP) under 1×CO₂ (solid) and 2×CO₂ (dashed). Data are masked using the three-step procedure described in the Methods. Rainfall increases across the entire PP under 2×CO₂, remaining elevated into autumn.

##### Supplementary Figure S8: Regional daily rainfall distributions (mm/day) and extreme-event statistics under 1×CO₂ (≈ 410 ppm; left) and 2×CO₂ (≈ 840 ppm; right) steady-state simulations. Results are shown for nine Arctic regions, each based on 400-year time slices. Grey dots show all daily values; coloured dots (blue/orange) mark days exceeding the monthly 99th percentile from the 1× baseline. Vertical black lines indicate the PP. Grey bars (right axis) show the proportion of days above threshold. Under 2×CO₂, extremes increase in all regions, though the seasonal timing and intensity vary.

##### Supplementary Figure S9: Regional return levels of daily rainfall extremes under 1×CO₂ (blue) and 2×CO₂ (orange) steady-state simulations. Results are based on 400-year time slices and calculated empirically from annual maximum daily precipitation (mm/day) during April–October. Return levels are consistently higher under 2×CO₂, but the degree of intensification differs between regions.

##### Supplementary Figure S10: Probability distributions of the number of extreme rainfall events experienced over a bird’s lifetime, modeled with a Poisson distribution. Left: lifespans of 10, 20, and 30 years with a return period of 9 years (λ ≈ 1.1, 2.2, 3.3). Right: lifespans of 10, 20, and 30 years with a return period of 2 years (λ = 5, 10, 15). Longer lifespans or shorter return periods increase both the expected number of events and the spread of the distribution.
